# Association of Primary Treatment Modality for Advanced-Stage Oropharyngeal Squamous Cell Carcinoma With Survival Outcomes

**DOI:** 10.1001/jamanetworkopen.2021.12067

**Published:** 2021-06-01

**Authors:** Mu-Hung Tsai, Yung-Jen Cheng, Tzu-Hui Pao, Wei-Ting Hsueh, Helen H.W. Chen, Yuan-Hua Wu

**Affiliations:** 1 Department of Radiation Oncology, National Cheng Kung University Hospital, College of Medicine, National Cheng Kung University, Tainan, Taiwan; 2 Institute of Computer Science and Information Engineering, National Cheng Kung University, Tainan, Taiwan

## Abstract

**Importance:**

Definitive chemoradiotherapy and upfront surgical treatment are both accepted as the standard of care for advanced-stage oropharyngeal squamous cell carcinoma. However, the optimal primary treatment modality remains unclear.

**Objective:**

To evaluate the comparative effectiveness of definitive chemoradiotherapy and upfront surgical treatment for advanced-stage oropharyngeal cancer.

**Design, Setting, and Participants:**

This retrospective comparative effectiveness analysis used data from the population-based Taiwan Cancer Registry. Included patients were diagnosed with clinical stage III or IV oropharyngeal squamous cell carcinoma from 2007 to 2015 and were identified from the registry. Patients with T4b or N3 disease were excluded. Data were analyzed from June 2019 through December 2020.

**Interventions:**

Definitive chemoradiotherapy or upfront surgical treatment.

**Main Outcomes and Measures:**

The primary outcome was overall survival, for which data were available through December 31, 2018. Secondary outcomes were progression-free survival, locoregional recurrence–free survival, and distant metastasis–free survival.

**Results:**

Among 1180 patients, 694 patients (58.8%) were in the definitive chemoradiotherapy group and 486 patients (41.2%) were in the upfront surgical treatment group. The median (interquartile range) follow-up was 3.62 (1.63-5.47) years, and most patients were men (1052 [89.1%] men) with a primary tumor in the tonsils (712 patients [60.3%]), moderately differentiated histology (604 patients [51.2%]), clinical N2 disease (858 patients [72.7%]), and clinical stage IVA disease (938 patients [79.5%]). The mean (SD) age was 54.59 (10.35) years. Primary treatment with an upfront surgical procedure was associated with a decreased risk of death during the study period (hazard ratio [HR], 0.81; 95% CI, 0.69-0.97; *P* = .02). However, when adjusted for age, subsite, histological grade, and T and N classification, upfront surgical treatment was no longer associated with an increased risk of death during the study period (HR, 0.96; 95% CI, 0.80-1.16; *P* = .70). Progression-free survival was worse in the group receiving upfront surgical treatment than in the group receiving chemoradiotherapy (HR, 1.64; 95% CI, 1.09-2.46; *P* = .02), and this difference persisted after adjusting for other factors associated with prognosis (ie, age, tumor subsite, histological grade, and T and N classification) (HR, 1.72; 95% CI, 1.12-2.66; *P* = .01).

**Conclusions and Relevance:**

This study found that definitive chemoradiotherapy was associated with effectiveness that was comparable with that of upfront surgical treatment when adjusted for baseline factors associated with prognosis. These findings suggest that definitive chemoradiotherapy should be considered to avoid accumulating toxic effects associated with surgical treatment and chemoradiotherapy.

## Key Points

**Question:**

What is the optimal primary treatment modality for patients with advanced-stage oropharyngeal squamous cell carcinoma?

**Findings:**

In this comparative effective research study among 1180 patients with advanced-stage oropharyngeal squamous cell carcinoma, primary treatment with definitive chemoradiotherapy vs upfront surgical treatment was not associated with a significant increase in the risk of death during the study period when adjusted for baseline factors associated with prognosis.

**Meaning:**

In this study, definitive chemoradiotherapy was associated with outcomes comparable with those of primary surgical treatment without accumulating toxic effects associated with surgical treatment and chemoradiotherapy.

## Teaser

This comparative effectiveness research investigates survival outcomes for chemoradiotherapy vs surgical treatment among patients with advanced-stage oropharyngeal squamous cell carcinoma.

## Introduction

The optimal primary treatment modality for advanced-stage oropharyngeal squamous cell carcinoma (OPSCC) is unclear. Definitive chemoradiotherapy and upfront surgical procedures are mainstream options in current treatment guidelines.^1^ Patients treated with upfront surgical procedures may require adjuvant therapy, such as radiotherapy (RT) or chemoradiotherapy, if adverse pathological features are found. The multimodal combination of surgical treatment and adjuvant RT or chemoradiotherapy is associated with increased incidence and severity of treatment-associated toxic effects and a poorer quality of life compared with surgical treatment alone or definitive chemoradiotherapy.^2,3^

Upfront surgery and definitive chemoradiotherapy resulted in comparable overall survival (OS) rates in patients with cT1-2N1-2b human papillomavirus (HPV)–negative OPSCC.^4^ Among patients treated with upfront surgical procedures, as many as 59.1% ultimately received trimodality treatment with adjuvant chemoradiotherapy. Another study^5^ of HPV-associated OPSCC found that upfront surgical treatment and chemoradiotherapy were associated with similar 3-year OS outcomes. Most patients who underwent surgical treatment (65.4%) also received adjuvant chemoradiotherapy. Irrespective of HPV status, these studies found similar outcomes among patients treated with upfront surgical procedures and those receiving definitive chemoradiotherapy, with more than half of patients in the upfront surgical treatment group undergoing trimodality treatment.^4,5^

Adjuvant treatment is frequently indicated after surgical treatment for patients with clinical stage III or nonmetastatic stage IV disease. Although this radical approach aims to achieve the maximum degree of cancer control, late sequelae are a concern following trimodality treatment. Thus, more information is needed on disease outcomes and treatment-associated toxic effects to guide therapeutic decision-making. Current literature is primarily restricted to early-stage disease. Nevertheless, the optimal treatment strategy remains unclear. Additionally, different oropharyngeal subsites (eg, the tonsils, base of the tongue, and soft palate) may also influence the choice of treatment.

The Taiwan Cancer Registry (TCR) collects data from all major hospitals and captures an estimated more than 98% of patients in Taiwan with newly diagnosed oropharyngeal cancer. This database contains detailed information on tumor location, tumor stage, treatment modalities, and recurrence.^6^ In this study, we compared the effectiveness associated with upfront surgical treatment and definitive chemoradiotherapy in treating patients with advanced-stage OPSCC.

## Methods

We followed the International Society for Pharmacoeconomics and Outcomes Research (ISPOR) reporting guideline for reporting comparative effectiveness analyses using secondary data sources. A certificate of exempt review and a waiver for informed consent were issued by the institutional review board of the National Cheng Kung University Hospital because the analysis was conducted using a deidentified database.

### Data Sources and Reporting

This comparative effectiveness research was performed using data from the Health and Welfare Data Science Center of Taiwan. Specifically, we used information from that center’s TCR and Cause of Death databases. The Health and Welfare Data Science Center allowed us to link records from the 2 databases using an anonymized but common identifier.

### Study Population

Patients aged 20 years or older without a history of malignant neoplasms were initially selected. Those with invasive squamous cell carcinoma (*International Classification of Diseases for Oncology, Third Revision* [*ICD-O-3*] codes M-8010, M-8070, and M-8071) in the base of the tongue (*ICD-O-3* site C01), tonsils (C09), or unspecified oropharyngeal sites (C10) were identified. We selected patients with advanced-stage nonmetastatic (ie, clinical stage III or IV) disease based on the seventh edition of the American Joint Committee on Cancer staging system. Patients with T4b or N3 disease (and soft palate or uvula primary sites) were excluded because such tumors are considered unresectable, and therefore, clinical equipoise between surgical treatment and chemoradiotherapy cannot be achieved.

Patients who underwent pharyngectomy with or without laryngectomy (surgical treatment codes 30-49) as their first treatment were assigned to the upfront surgical treatment group, whereas patients who received concurrent chemoradiotherapy (of ≥60 Gy) were assigned to the chemoradiotherapy group. Patients receiving neoadjuvant chemotherapy, neoadjuvant RT, or sequential chemoradiotherapy were excluded because there is no well-established standard regimen in these scenarios. Patients treated with definitive RT alone were also excluded because such treatment is considered suboptimal for advanced-stage OPSCC.

### Patient Covariates and Outcomes

Data on age, sex, T and N classification, treatment modalities, and disease status at last follow-up were extracted from the TCR. Because the association of age with survival may not be linear, we analyzed age as a continuous variable using a restricted cubic spline method.

We defined OS as the time from the date of biopsy to the date of death. Data were obtained from the Cause of Death database. If no record of death was found, the patient was assumed to be alive and censored on the last day of available database records (ie, December 31, 2018). Progression-free survival (PFS) was calculated from the date of biopsy to the date of any recurrence. Locoregional recurrence–free survival and distant metastasis–free survival were calculated from the date of biopsy to the date of locoregional recurrence or distant metastasis, respectively. We calculated PFS, locoregional recurrence–free survival, and distant metastasis–free survival based solely on data in the TCR.

### Statistical Analysis

Baseline demographic characteristics and T and N classifications were compared using χ^2^ tests. The Kruskal-Wallis test or *t* tests were used to compare continuous variables. Univariable analysis was performed by plotting Kaplan-Meier survival curves and using the log-rank test. The independent effects of prognostic factors were evaluated using a multivariable Cox proportional hazards regression model. Known prognostic factors were included in the model: age, sex, primary tumor subsite, histological grade, clinical T and N classification, and primary treatment modality. An exploratory subgroup analysis was performed to estimate the association of primary treatment modality with the risk of death for each primary subsite (ie, tonsils, base of tongue, and unspecified oropharyngeal subsites). To improve robustness, we used a propensity score–matched cohort design to reduce the differences in baseline characteristics between the surgical treatment and chemoradiotherapy groups. A multivariable logistic regression model, including age, sex, primary tumor subsite, histological grade, and clinical T and N classification, was used to generate propensity scores. One-to-one matching was performed using nearest neighbor matching without replacement. Finally, landmark analysis was performed to assess the effect of survival bias (immortal time bias). We conducted 3 separate analyses restricted to patients who survived more than 12 months, more than 18 months, and more than 24 months. All statistical analyses were conducted using R statistical software version 3.6.3 (R Project for Statistical Computing) from June 2019 through December 2020. A 2-sided *P* < .05 was considered statistically significant. Hazard ratios (HRs) and 95% CIs were also reported.

## Results

### Baseline Patient Characteristics

Among 1180 patients diagnosed with advanced-stage OPSCC from 2007 through 2015 who met inclusion criteria, most patients were men (1052 [89.1%] men) with a primary tumor located in the tonsils (712 individuals [60.3%]), moderately differentiated histology (604 individuals [51.2%]), and clinical N2 disease (858 individuals [72.7%]) (Table 1). The mean (SD) age was 54.59 (10.35) years. There were 242 patients (20.51%) with stage III disease and 938 patients (79.5%) with stage IVA disease. There were 184 patients (15.6%) with clinical T1 disease, 466 patients (39.5%) with clinical T2 disease, 221 patients (18.7%) with clinical T3 disease, and 309 patients (26.2%) with clinical T4a disease. The primary treatment modality was definitive chemoradiotherapy for 694 patients (58.8%) and upfront surgical treatment for 486 patients (41.2%). The median (interquartile range [IQR]) follow-up was 3.62 (1.63-5.47) years for the entire study group and 5.13 (3.93-6.49) years for surviving patients. Patients in the chemoradiotherapy group, compared with those in the upfront surgical treatment group, were statistically significantly older (mean [SD], 55.36 [10.67] years vs 53.49 [9.78] years; *P* = .003) and had a statistically significantly larger median (IQR) tumor size (39.00 [28.00-55.00] mm vs 30.00 [20.25-44.00] mm; *P* < .001) and more advanced T stage (eg, 198 [28.5%] individuals vs 111 [22.8%] individuals with T4a disease; *P* < .001). The oropharyngeal subsite was imbalanced between the groups. More patients had lesions at the base of the tongue in the chemoradiotherapy group than in the upfront surgical treatment group. Conversely, more patients had tonsillar lesions in the upfront surgical treatment group than in the chemoradiotherapy group. In the upfront surgical treatment group, 427 patients (87.9%) received adjuvant RT with or without concurrent chemotherapy. Conversely, 20 patients (2.9%) underwent salvage surgical treatment in the chemoradiotherapy group.

**Table 1.** Patient and Tumor Characteristics

Characteristic	Patients by primary treatment modality, No. (%)		*P* value
	Chemoradiotherapy (n = 694)	Surgical treatment (n = 486)	
Sex			
Men	624 (89.9)	428 (88.1)	.37
Women	70 (10.1)	58 (11.9)	
Age, mean (SD), y	55.36 (10.67)	53.49 (9.78)	.003
Subsite			
Tonsil	394 (56.8)	318 (65.4)	.01
Base of tongue	209 (30.1)	116 (23.9)	
Oropharynx, not otherwise specified	91 (13.1)	52 (10.7)	
Histological grade			
Well differentiated (grade 1)	20 (2.9)	25 (5.1)	<.001
Moderately differentiated (grade 2)	327 (47.1)	277 (57.0)	
Poorly differentiated (grades 3-4)	173 (24.8)	150 (30.9)	
Unknown	174 (25.1)	34 (7.0)	
Tumor size, median (IQR), mm	39.00 (28.00-55.00)	30.00 (20.25-44.00)	<.001
Clinical T classification			
T1	78 (11.2)	106 (21.8)	<.001
T2	260 (37.5)	206 (42.4)	
T3	158 (22.8)	63 (13.0)	
T4a	198 (28.5)	111 (22.8)	
Clinical N classification			
N0	54 (7.8)	33 (6.8)	.08
N1	123 (17.7)	112 (23.0)	
N2	517 (74.5)	341 (70.2)	
Clinical stage			
III	137 (19.7)	105 (21.6)	.48
IVA	557 (80.3)	381 (78.4)	
Radiotherapy dose, median (IQR), Gy	70 (70-72)	66 (60-70)^a^	<.001
Radiotherapy fractions, median (IQR), No.	35 (35-36)	33 (30-35)^a^	<.001
Adjuvant treatment after primary treatment			
None	674 (97.1)	59 (12.1)	NA
Salvage neck dissection	11 (1.6)	NA	
Salvage surgical treatment	9 (1.3)	NA	
RT alone	NA	188 (38.7)	
Chemoradiotherapy	NA	239 (49.2)	
Follow-up, median (SD), y	3.55 (2.18)	3.82 (2.19)	.03

Abbreviations: IQR, interquartile range; NA, not applicable; RT, radiotherapy.

^a^ Calculated for patients receiving adjuvant RT.

### Independent Variables Associated With OS

**Figure 1.**
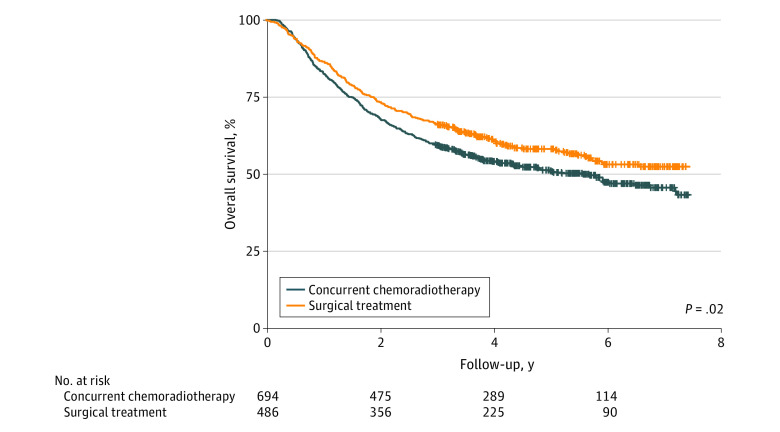
Kaplan-Meier Curves for Overall Survival

The 3-year OS rate was 66.3% (95% CI, 62.2%-70.6%) for patients who underwent upfront surgical treatment and 59.3% (95% CI, 55.7%-63.0%) for patients who received chemoradiotherapy. Compared with definitive chemoradiotherapy, upfront surgical treatment as the primary treatment modality was associated with decreased risk of death during the study period (HR, 0.81; 95% CI, 0.69-0.97; *P* = .02) (Figure 1). Female sex, tonsillar primary site, poorly differentiated histology, and clinical N1 disease were also associated with a statistically significantly decreased risk of death during the study period. However, in a multivariable analysis adjusting for age, tumor subsite, histological grade, and T and N classification, primary treatment modality was not associated with a statistically significant difference in risk of death (HR, 0.96; 95% CI, 0.80-1.16; *P* = .70) (Table 2). Male sex, older age, nontonsillar primary sites, and T3 through T4 N2 disease were independent variables associated with worse OS.

**Table 2.** Univariable and Multivariable Cox Proportional Hazards Model for Overall Survival Among 1180 Patients

Variable	Univariable		Multivariable	
	HR (95% CI)	*P* value	HR (95% CI)	*P* value
Sex				
Male	1 [Reference]	NA	1 [Reference]	NA
Female	0.21 (0.13-0.34)	<.001	0.27 (0.17-0.44)	<.001
Subsite				
Tonsil	1 [Reference]	NA	1 [Reference]	NA
Base of tongue	2.03 (1.69-2.44)	<.001	1.45 (1.18-1.77)	<.001
Oropharynx, not otherwise specified	1.85 (1.45-2.36)	<.001	1.43 (1.11-1.84)	.006
Histological grade				
Well differentiated (grade 1)	1.20 (0.81-1.78)	.36	0.93 (0.62-1.39)	.72
Moderately differentiated (grade 2)	1 [Reference]	NA	1 [Reference]	NA
Poorly differentiated (grades 3-4)	0.64 (0.52-0.80)	<.001	0.77 (0.62-0.97)	.02
Unknown	1.03 (0.83-1.28)	.80	1.02 (0.82-1.28)	.85
Clinical T classification				
T1	1 [Reference]	NA	1 [Reference]	NA
T2	1.24 (0.93-1.65)	.14	1.15 (0.86-1.53)	.35
T3	1.66 (1.22-2.27)	.001	1.45 (1.05-2.02)	.03
T4a	2.71 (2.04-3.60)	<.001	1.98 (1.46-2.69)	<.001
Clinical N classification				
N0	1 [Reference]	NA	1 [Reference]	NA
N1	0.83 (0.58-1.19)	.30	1.28 (0.88-1.87)	.2
N2	0.98 (0.71-1.36)	.93	1.48 (1.06-2.08)	.02
Primary treatment modality				
Concurrent chemoradiotherapy	1 [Reference]	NA	1 [Reference]	NA
Surgical treatment	0.81 (0.69-0.97)	.02	0.96 (0.80-1.16)	.70

Abbreviations: HR, hazard ratio; NA, not applicable.

### Subsite Analysis

Oropharyngeal tumors arising from nontonsillar regions were associated with statistically significantly worse OS than tonsillar tumors, a difference that remained significant when adjusted for age, histological grade, and T and N classification (Table 2). An exploratory analysis was performed to determine the prognostic significance associated with oropharyngeal subsite and the relative effectiveness associated with primary treatment modality for different subsites.

In a subgroup analysis of primary treatment modality for different subsites, we found no association between primary treatment modality and risk of death during the study period in either subgroup (tonsils: HR, 0.90; 95% CI, 0.70-1.14; *P* = .38; base of the tongue: HR, 0.82; 95% CI, 0.61-1.11; *P* = .19; unspecified oropharyngeal subsites: HR, 0.83; 95% CI, 0.53-1.30; *P* = .41). This is consistent with the results of our primary analysis.

### PFS and Pattern of Failure

**Figure 2.**
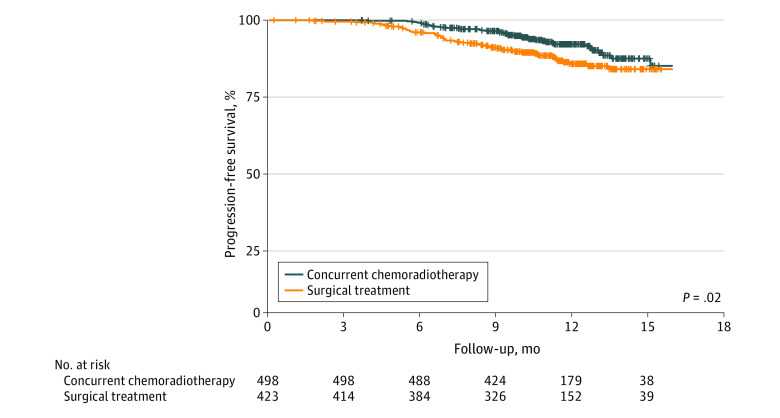
Kaplan-Meier Curves for Progression-Free Survival

Recurrence data were available for 922 of 1180 patients (78.1%) included in this study, including 498 patients in the chemoradiotherapy group (71.8%) and 423 patients in the upfront surgical treatment group (87.0%). The median (IQR) follow-up for recurrence was 11.1 (9.5-12.7) months. The upfront surgical treatment group had statistically significantly worse PFS than the chemoradiotherapy group (HR, 1.64; 95% CI, 1.09-2.46; *P* = .02) (Figure 2). Patients in the chemoradiotherapy group were more likely to be progression free at 12 months than those in the upfront surgical treatment group (91.8% [95% CI, 89.1%-94.5%] vs 85.1% [95% CI, 81.3%-89.1%]). When the multivariable model was adjusted for age, tumor subsite, histological grade, and T and N classification, primary treatment modality remained associated with statistically significantly worse PFS (HR, 1.72; 95% CI, 1.12-2.66; *P* = .01) (eTable 1 in the Supplement). Additional independent variables associated with disease recurrence included nontonsillar primary sites and clinical N2 disease.

**Figure 3.**
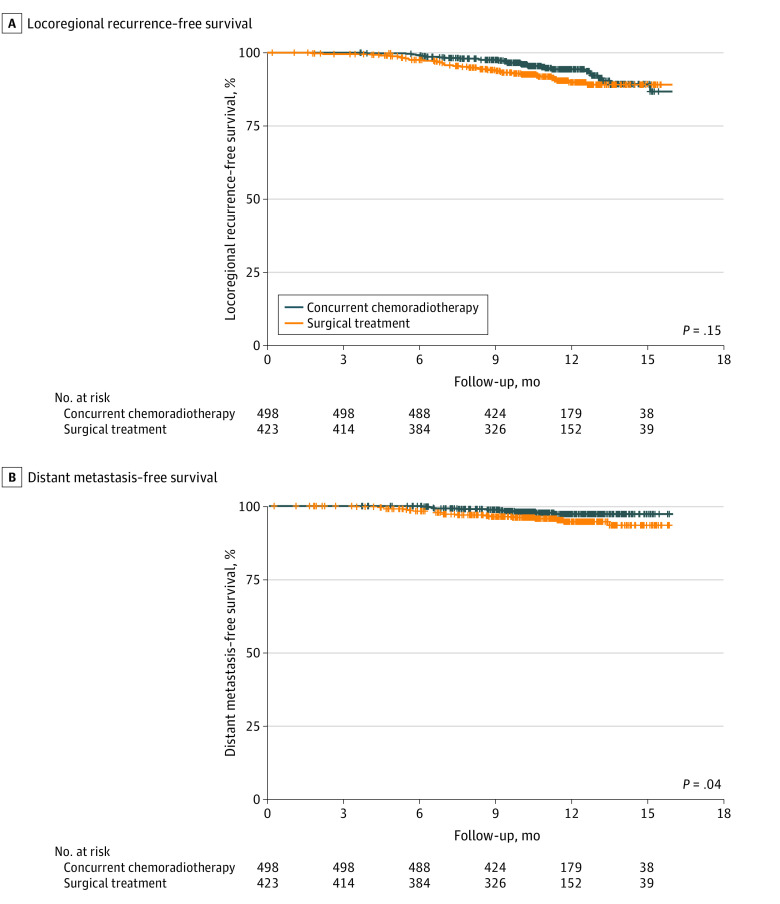
Kaplan-Meier Curves for Locoregional Recurrence–Free Survival and Distant Metastasis–Free Survival

There was no statistically significant difference in locoregional recurrence–free survival between the chemoradiotherapy and upfront surgical treatment groups (Figure 3A). Conversely, there was a significant improvement in distant metastasis–free survival in the chemoradiotherapy group (Figure 3B). Locoregional recurrence was documented as the first site of relapse in 30 patients (6.0%) in the chemoradiotherapy group and 35 patients (8.3%) in the upfront surgical treatment group. In contrast, 11 patients (2.2%) in the chemoradiotherapy group and 19 patients (4.5%) in the upfront surgical group developed distant metastases.

### Propensity Score–Matched Group

We performed a propensity-matched comparison to reduce imbalances between the groups. Propensity matching resulted in 2 groups of 486 patients with balanced characteristics (ie, age, subsite, histological grade, and N classification). There was residual imbalance in T classification (eTable 2 in the Supplement).

In the propensity score–matched group, there was no significant difference in the risk of death during the study period between the upfront surgical treatment and chemoradiotherapy groups (HR, 0.88; 95% CI, 0.73-1.07; *P* = .19) (eTable 3 in the Supplement). Female sex, tonsillar primary site, and poorly differentiated histology were associated with a decreased risk of death during the study period. In the multivariable analysis adjusted for age, subsite, histological grade, and T and N classification, primary treatment modality was not associated with a statistically significant difference in the risk of death during the study period (HR, 0.95; 95% CI, 0.78-1.14; *P* = .57). Male sex, nontonsillar primary sites, and T3 through T4 N2 disease were independent variables associated with statistically significantly worse OS in the propensity score–matched group.

### Landmark Analysis

Finally, we performed a landmark analysis of patients who survived for more than 12 months, more than 18 months, and more than 24 months (eFigure in the Supplement). Consistent with the results of our primary analysis, similar survival rates were observed in the chemoradiotherapy and upfront surgical treatment groups.

## Discussion

In this comparative effectiveness research, we found that definitive chemoradiotherapy was associated with similar survival rates as upfront surgical treatment in patients with advanced-stage OPSCC after adjusting for baseline prognostic factors. A greater proportion of patients in the chemoradiotherapy group were progression free, owing to the lower risk of distant metastasis. Our results are consistent with those of previous studies,^4,5^ with the added benefit of showing comparable disease control in the primary treatment modalities.

Multimodal therapy is expensive and can potentially lead to morbidity, which may impair the patient’s quality of life. In early-stage OPSCC, surgical treatment alone can provide effective disease control and limit long-term sequelae. In a retrospective study,^7^ transoral robotic surgical treatment was associated with decreased costs and increased quality-adjusted life-years compared with chemoradiotherapy. However, this advantage diminished with increasing adjuvant therapy after transoral robotic surgical treatment. Two large-scale observational studies^4,5^ compared the effectiveness of upfront surgical treatment and chemoradiotherapy in patients with T1 through T2, N1 through N2b OPSCC. Similar survival rates were reported in both groups, irrespective of HPV status. Notably, 59% to 65% of patients who underwent upfront surgical treatment also required adjuvant chemoradiotherapy.

Most patients with advanced-stage OPSCC require adjuvant RT or chemoradiotherapy following upfront surgical treatment. In this study, more than 80% of patients who underwent upfront surgical treatment were also treated with adjuvant RT or chemoradiotherapy. This multimodal treatment strategy may result in organ dysfunction and late severe neck fibrosis, especially in patients requiring extensive surgical treatment with flap reconstruction.^2,8,9^ Therefore, efforts should be made to avoid trimodality treatment without compromising disease control. Sher et al^10^ compared the effectiveness associated with primary RT and surgical treatment in older patients with OPSCC. The authors selected 2754 patients aged 66 years or older with advanced-stage OPSCC from the Surveillance, Epidemiology, and End Results–Medicare linked database. Most patients (69%) were treated with radiation-based therapy, while 31% of the patients underwent primary surgical treatment. The 3-year OS rates were similar between the primary RT and primary surgical treatment groups (52.8% vs 54.9%).

A 2019 study^11^ investigated intensive trimodality treatment for patients with advanced-stage OPSCC. This study focused on patients with T3 to T4, N2 to N3 disease, as defined by the seventh edition of the American Joint Committee on Cancer staging system. Compared with chemoradiotherapy, trimodality treatment was not associated with statistically significant differences in prolonged OS or cause-specific survival in patients with HPV-negative OPSCC. Meanwhile, trimodal therapy was associated with a benefit in OS among only a subset of patients with advanced-stage HPV-positive OPSCC. After adjusting for known prognostic factors (eg, age, sex, ethnicity, and smoking status), the benefit of trimodality treatment was significant in the N2 through N3 subgroup for OS and cancer-specific survival. However, these results do not conflict with our findings because HPV-positive disease is found in a small proportion of Taiwanese patients with oropharyngeal cancer, and patients with N3 disease were excluded from our study. Further studies are needed to identify patients who may benefit from trimodality treatment.

In this study, we reported a favorable PFS in patients treated with chemoradiotherapy owing to the lower rate of distant metastasis. This observation may be explained by the exposure to chemotherapy for patients in the chemoradiotherapy group. Among patients who underwent upfront surgical treatment, 49.2% received chemotherapy, while all patients in the chemoradiotherapy group received concurrent chemotherapy. A population-based study^12^ in Denmark investigated predictive factors associated with survival and failure patterns in 1244 patients with OPSCC. According to the results, 16% of patients had locoregional recurrence, while 7% of patients had distant recurrence. Risk factors associated with distant recurrence included sex, T or N classification, and HPV status. Our study focused on patients with advanced T and N stage disease, who were at a higher risk of distant recurrence.

The oropharyngeal subsite may also influence treatment decisions. Chemoradiotherapy is the preferred treatment modality in the interest of organ preservation for lesions at the base of the tongue compared with other subsites, such as the tonsils. In our study, a greater proportion of patients had lesions at the base of the tongue in the chemoradiotherapy group than in the upfront surgical treatment group (30.1% vs 23.9%). Culié et al^13^ conducted a multicenter study to identify clinical factors that may influence therapeutic decision making in 476 patients with OPSCC, in which 244 patients (51%) had HPV-positive disease. The primary tumor location at the glossotonsillar sulcus was one of the main reasons for the preference for upfront surgical treatment. More patients with HPV-positive OPSCC underwent nonsurgical treatment than patients with HPV-negative OPSCC, although this difference was not statistically significant. Sher et al^10,14^ found that tonsillar cancers were associated with better survival rates than cancers of the base of the tongue. Tham et al^15^ found that in patients with HPV-positive disease, nontonsillar subsites were associated with statistically significantly worse cause-specific survival than tonsillar subsites (HR, 2.16; 95% CI, 1.20-3.86; *P* = .01). Our results are consistent with previous study results in finding that cancers of the base of the tongue are associated with worse survival and shorter PFS rates. Despite this, we observed similar survival rates in the chemoradiotherapy and upfront surgical treatment groups for each subsite in the subgroup analysis.

### Limitations

This study has several limitations. First, the retrospective design may have led to differences between the groups in potential confounding variables, such as comorbidities, performance status, and socioeconomic status. Second, certain prognostic factors, such as HPV status, are not mandated in the registry because the proportion of oropharyngeal cancers in Taiwan that are HPV-related is low.^16^ However, in 2 previous National Cancer Database studies, comparable outcomes were observed between upfront surgical treatment and definitive chemoradiotherapy in patients with HPV-positive disease^5^ and those with HPV-negative disease,^4^ suggesting that HPV status may not be associated with the comparative effectiveness of these primary treatment modalities. Further studies are needed to define patients with T3 to T4 OPSCC who may benefit from trimodality treatment, separately for patients with HPV-positive and HPV-negative disease. Third, the median follow-up for recurrence in the TCR was only 11.1 months. Nevertheless, despite the short follow-up time, these data on disease control remain invaluable. They provide insight into the pattern of failure, which is not available from the National Cancer Database or Surveillance, Epidemiology, and End Results registry.

## Conclusions

This study provides comprehensive data on disease outcomes, failure patterns, and adjuvant treatment associated with different primary modalities in a predominantly HPV-negative population. We found comparable effectiveness in the 2 primary treatment modalities when adjusted for baseline prognostic factors. We also found that these results were consistent between subgroups of patients with different OPSCC subsites. These findings suggest that definitive chemoradiotherapy should be considered first given that it may prevent the accumulation of surgical treatment and chemoradiotherapy toxic effects in patients with advanced-stage OPSCC. Our findings suggest that upfront surgical treatment should be used with discretion in patients with advanced-stage OPSCC if further adjuvant chemoradiotherapy is anticipated.

## Notes

Supplement. **eTable 1.** Univariable and Multivariable Cox Proportional Hazards Model for Progression-Free Survival (n = 922) **eTable 2.** Patient and Tumor Characteristics of the Propensity-Matched Group (n = 972) **eTable 3.** Univariable and Multivariable Cox Proportional Hazards Model for Overall Survival in the Propensity-Matched Group (n = 972) **eFigure.** Landmark Analysis Restricted to Patients Who Survived for More Than 12, More Than 18, and More Than 24 Months
