# Supplementary material for: Association of Primary Treatment Modality for Advanced-Stage Oropharyngeal Squamous Cell Carcinoma With Survival Outcomes
Source: JAMA Netw Open. 2021 Jun 1;4(6):e2112067. doi: 10.1001/jamanetworkopen.2021.12067 (PMC8170546; doi:10.1001/jamanetworkopen.2021.12067)
Supplement: Supplement. — eTable 1. Univariable and Multivariable Cox Proportional Hazards Model for Progression-Free Survival (n = 922) eTable 2. Patient and Tumor Characteristics of the Propensity-Matched Group (n = 972) eTable 3. Univariable and Multivariable Cox Proportional Hazards Model for Overall Survival in the Propensity-Matched Group (n = 972) eFigure. Landmark Analysis Restricted to Patients Who Survived for More Than 12, More Than 18, and More Than 24 Months [file jamanetwopen-e2112067-s001.pdf]

Supplemental Online Content

Tsai MH, Cheng YJ, Pao TH, Hsueh WT, Chen HHW, Wu YH. Association of primary treatment modality for advanced-stage oropharyngeal squamous cell carcinoma with survival outcomes. *JAMA Netw Open*. 2021;4(6):e2112067. doi:10.1001/jamanetworkopen.2021.12067

eTable 1. Univariable and Multivariable Cox Proportional Hazards Model for Progression-Free Survival (n = 922)
eTable 2. Patient and Tumor Characteristics of the Propensity-Matched Group (n = 972)
eTable 3. Univariable and Multivariable Cox Proportional Hazards Model for Overall Survival in the Propensity-Matched Group (n = 972)
eFigure. Landmark Analysis Restricted to Patients Who Survived for More Than 12, More Than 18, and More Than 24 Months

This supplemental material has been provided by the authors to give readers additional information about their work.

© 2021 Tsai MH et al. *JAMA Network Open.*

eTable 1. Univariable and Multivariable Cox Proportional Hazards Model for Progression-Free Survival (n = 922)

	Univariable		Multivariable	
Variable	Hazard ratio (95% CI)	p-value	Hazard ratio (95% CI)	p-value
Sex				
Male	Reference		Reference	
Female	0.30 (0.11-0.81)	0.02	0.43 (0.15-1.21)	0.11
Subsite				
Tonsil	Reference		Reference	
Base of tongue	2.29 (1.46-3.60)	<0.001	2.27 (1.39-3.71)	0.001
Oropharynx, NOS	2.88 (1.67-4.96)	<0.001	3.26 (1.82-5.84)	<0.001
Histological grade				
Well-differentiated (Grade 1)	1.68 (0.72-3.90)	0.23	1.25 (0.52-2.98)	0.62
Moderately differentiated (Grade 2)	Reference		Reference	
Poorly differentiated (Grade 3-4)	0.69 (0.43-1.13)	0.14	0.87 (0.53-2.98)	0.60
Unknown	0.53 (0.27-1.05)	0.07	0.58 (0.29-1.16)	0.12
Clinical T classification				
T1	Reference		Reference	
T2	1.25 (0.63-2.47)	0.52	1.28 (0.64-2.56)	0.48
T3	1.63 (0.78-3.43)	0.20	1.67 (0.76-3.67)	0.20
T4a	2.29 (1.15-4.57)	0.02	1.63 (0.78-3.39)	0.19
Clinical N classification				
N0	Reference		Reference	
N1	1.26 (0.42-3.81)	0.68	1.85 (0.59-5.80)	0.29
N2	1.98 (0.72-5.41)	0.18	3.49 (1.23-9.86)	0.02
Primary Treatment Modality				
CCRT	Reference		Reference	
Surgery	1.64 (1.09-2.46)	0.02	1.72 (1.12-2.66)	0.01

Abbreviations: CI, confidence interval; NOS, not otherwise specified; CCRT, concurrent chemoradiotherapy

© 2021 Tsai MH et al. *JAMA Network Open.*

eTable 2. Patient and Tumor Characteristics of the Propensity-Matched Group (n = 972)

**Primary Treatment Modality**	**Chemoradiotherapy (n = 486)**	**Surgery (n = 486)**	***p*-value**
Sex, n (%)			0.61
Male	434 (89.3)	428 (88.1)	
Female	52 (10.7)	58 (11.9)	
Age, mean (SD), years	53.59 (9.11)	53.49 (9.78)	0.27
Subsite, n (%)			
Tonsil	295 (60.7)	318 (65.4)	
Base of tongue	137 (28.2)	116 (23.9)	
Oropharynx, NOS	54 (11.1)	52 (10.7)	
Histological grade			0.47
Well-differentiated (Grade 1)	18 (3.7)	25 (5.1)	
Moderately differentiated (Grade 2)	291 (59.9)	277 (57.0)	
Poorly differentiated (Grade 3-4)	137 (28.2)	150 (30.9)	
Unknown	40 (8.2)	34 (7.0)	
Clinical T classification			0.001
T1	65 (13.4)	106 (21.8)	
T2	197 (40.5)	206 (42.4)	
T3	79 (16.3)	63 (13.0)	
T4a	145 (29.8)	111 (22.8)	
Clinical N classification			0.36
N0	36 (7.4)	33 (6.8)	
N1	94 (19.3)	112 (23.0)	
N2	356 (73.3)	341 (70.2)	

Abbreviations: SD, standard deviation; NOS, not otherwise specified

© 2021 Tsai MH et al. *JAMA Network Open.*

eTable 3. Univariable and Multivariable Cox Proportional Hazards Model for Overall Survival in the Propensity-Matched Group (n = 972)

	**Univariable**		**Multivariable**	
**Variable**	**Hazard ratio (95% CI)**	***p*-value**	**Hazard ratio (95% CI)**	***p*-value**
Sex				
Male	Reference		Reference	
Female	0.16 (0.09-0.29)	<0.001	0.22 (0.12-0.39)	<0.001
Subsite				
Tonsil	Reference		Reference	
Base of tongue	2.04 (1.66-2.51)	<0.001	1.52 (1.21-1.91)	<0.001
Oropharynx, NOS	1.97 (1.50-2.61)	<0.001	1.54 (1.15-2.06)	0.004
Histological grade				
Well-differentiated (Grade 1)	1.25 (0.83-1.88)	0.29	0.97 (0.64-1.47)	0.88
Moderately differentiated (Grade 2)	Reference		Reference	
Poorly differentiated (Grade 3-4)	0.60 (0.48-0.76)	<0.001	0.73 (0.57-0.93)	0.01
Unknown	0.96 (0.68-1.36)	0.84	1.01 (0.71-1.44)	0.95
Clinical T classification				
T1	Reference		Reference	
T2	1.35 (0.99-1.84)	0.06	1.29 (0.94-1.76)	0.12
T3	1.88 (1.32-2.70)	<0.001	1.61 (1.10-2.35)	0.02
T4a	2.83 (2.07-3.86)	<0.001	2.04 (1.46-2.85)	<0.001
Clinical N classification				
N0	Reference		Reference	
N1	0.83 (0.55-1.24)	0.36	1.35 (0.88-2.07)	0.17
N2	0.93 (0.65-1.35)	0.72	1.51 (1.03-2.22)	0.04
Primary Treatment Modality				
CCRT	Reference		Reference	
Surgery	0.88 (0.73-1.07)	0.19	0.95 (0.78-1.14)	0.57

Abbreviations: CI, confidence interval; NOS, not otherwise specified; CCRT, concurrent chemoradiotherapy

© 2021 Tsai MH et al. *JAMA Network Open.*

eFigure. Landmark Analysis Restricted to Patients Who Survived for More Than 12, More Than 18, and More Than 24 Months

CCRT, concurrent chemoradiotherapy.

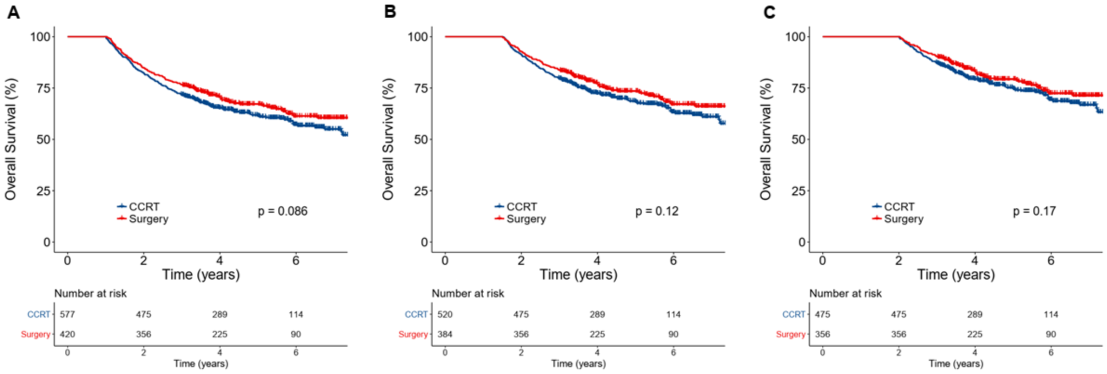

© 2021 Tsai MH et al. *JAMA Network Open.*
